# Tri-antennary tri-sialylated mono-fucosylated glycan of alpha-1 antitrypsin as a non-invasive biomarker for non-alcoholic steatohepatitis: a novel glycobiomarker for non-alcoholic steatohepatitis

**DOI:** 10.1038/s41598-019-56947-1

**Published:** 2020-01-15

**Authors:** Koji Ogawa, Takashi Kobayashi, Jun-ichi Furukawa, Hisatoshi Hanamatsu, Akihisa Nakamura, Kazuharu Suzuki, Naoki Kawagishi, Masatsugu Ohara, Machiko Umemura, Masato Nakai, Takuya Sho, Goki Suda, Kenichi Morikawa, Masaru Baba, Ken Furuya, Katsumi Terashita, Tomoe Kobayashi, Manabu Onodera, Takahiro Horimoto, Keisuke Shinada, Seiji Tsunematsu, Izumi Tsunematsu, Takashi Meguro, Tomoko Mitsuhashi, Megumi Hato, Kenichi Higashino, Yasuro Shinohara, Naoya Sakamoto

**Affiliations:** 1 Department of Gastroenterology and Hepatology, Graduate School of Medicine Hokkaido University, Sapporo, Japan; 2 Biomarker R&D Department, Shionogi & Co., Ltd., Osaka, Japan; 3 Department of Advanced Clinical Glycobiology, Graduate School of Medicine, Hokkaido University, Sapporo, Japan; 4 Department of Gastroenterology, Japan Community Health Care Organization (JCHO) Hokkaido Hospital, Sapporo, Japan; 5 Department of Internal Medicine, Kushiro Rosai Hospital, Kushiro, Japan; 6 Department of Gastroenterology, Tomakomai City Hospital, Tomakomai, Japan; 7 Department of Gastroenterology, NTT-East Sapporo Hospital, Sapporo, Japan; 8 Department of Gastroenterology, Aiiku Hospital, Sapporo, Japan; 9 Department of Gastroenterology, Keiwakai Ebetsu Hospital, Ebetsu, Japan; 10 Department of Gastroenterology, Hokkaido Medical Centre, Sapporo, Japan; 11 Department of Gastroenterology, Touei Hospital, Sapporo, Japan; 12 Department of Gastroenterology, Hokkaido Gastroenterology Hospital, Sapporo, Japan; 13 Department of Surgical Pathology, Graduate School of Medicine Hokkaido University, Sapporo, Japan; 14 Department of Pharmacy, Kinjo Gakuin University, Nagoya, Japan

**Keywords:** Diagnostic markers, Hepatology

## Abstract

Non-alcoholic steatohepatitis (NASH) is a progressive form of non-alcoholic fatty liver disease (NAFLD) that may lead to liver cirrhosis or hepatocellular carcinoma. Here, we examined the diagnostic utility of tri-antennary tri-sialylated mono-fucosylated glycan of alpha-1 antitrypsin (AAT-A3F), a non-invasive glycobiomarker identified in a previous study of NASH diagnosis. This study included 131 biopsy-proven Japanese patients with NAFLD. We evaluated the utility of AAT-A3F in NASH diagnosis, and conducted genetic analysis to analyse the mechanism of AAT-A3F elevation in NASH. Serum AAT-A3F concentrations were significantly higher in NASH patients than in NAFL patients, and in patients with fibrosis, lobular inflammation, and ballooning. Hepatic FUT6 gene expression was significantly higher in NASH than in NAFL. IL-6 expression levels were significantly higher in NASH than in NAFL and showed a positive correlation with FUT6 expression levels. The serum-AAT-A3F levels strongly correlated with hepatic FUT6 expression levels. AAT-A3F levels increased with fibrosis, pathological inflammation, and ballooning in patients with NAFLD and may be useful for non-invasive diagnosis of NASH from the early stages of fibrosis.

## Introduction

Non-alcoholic fatty liver disease (NAFLD) is a hepatic manifestation of metabolic syndrome that is frequently associated with obesity, diabetes mellitus, and dyslipidaemia, without a history of significant alcohol consumption^1^. NAFLD is sub-divided into non-alcoholic fatty liver (NAFL; a non-progressive form of NAFLD) and non-alcoholic steatohepatitis (NASH; a progressive form that can lead to cirrhosis and the development of hepatocellular carcinoma [HCC]^2–4^).

To date, liver biopsy remains the gold standard for NAFLD diagnosis and staging^5^. However, liver biopsy is an invasive procedure that may lead to undesirable complications^6,7^ and sampling bias. Several non-invasive biomarkers and scoring systems have been reported^8^, but no established diagnostic tests or biomarkers are available at present. Thus, identifying and validating novel non-invasive biomarkers would be valuable.

Glycosylation is one of the most common post-translational modifications occurring in proteins. Glycosylation can affect the biological activities of proteins, their transport towards the cell surface, and stabilization of their functional conformations^9^. Glycans on human glycoproteins are mainly composed of only 10 kinds of monosaccharides: glucose, galactose, mannose, *N*-acetylglucosamine, *N*-acetylgalactosamine, fucose, *N*-acetylneuraminic acid (a type of sialic acid), xylose, glucuronic acid, and iduronic acid. However, the order of sugars on glycans and the mode of linkage between each sugar result in high diversity of glycan structures. Glycans are the determinants of some clinically used cancer biomarkers such as CA19-9, CA125, and AFP-L3. Regarding liver diseases, glycosylation changes on immunoglobulins^10^, haptoglobin^11^, and alpha-1 antitrypsin^12^ were reported for patients with liver cirrhosis. Recently, mac-2-binding protein glycosylation isomer (M2BPGi) was reported as a novel fibrosis biomarker^13^ and has shown utility in NAFLD^14^. As represented by these molecules, glycosylation changes reflecting liver disease severity are now receiving increasing attention.

Previously, we determined the N-glycomic profile of focused serum glycoproteins from patients with NAFL or NASH by focused protein glycomic analysis (FPG), and successfully discovered several NASH biomarker candidates. Among them, tri-antennary tri-sialylated mono-fucosylated glycan of alpha-1 antitrypsin (AAT-A3F) showed the most dynamic change. In addition, a simplified method named immunoprecipitation glycomics (IPG), which employs affinity bead purification and matrix-assisted laser desorption/ionization–time-of-flight mass spectrometry (MALDI–TOF MS), was developed for the rapid determination of AAT-A3F^15^. The simplified method enabled us to quantitatively measure AAT-A3F in a high-throughput manner.

In this study, we investigated the diagnostic utility of AAT-A3F identified in our previous exploratory study on NASH biomarkers.

## Results

### Characteristics of the NAFLD patients

A total of 131 NAFLD patients (57 with NAFL, 74 with NASH) were enrolled in this study. Patients were divided into NAFL and NASH groups based on the pathological appearance of their liver biopsy specimens. The NASH group had a higher proportion of females than the NAFL group. Age, AST, HbA1c, FBS, and HOMA-IR were significantly higher in the NASH group than in the NAFL group. In contrast, albumin and LDL-C were significantly lower in the NASH group than in the NAFL group. BMI, Plt, ALT, AFP, and TG were not significantly different between the NAFL and NASH groups. Serum cCK18 and M2BPGi levels were significantly higher in the NASH group than in the NAFL group. Both fibrosis-prediction equations (FIB-4 index and APRI) were significantly higher in the NASH group than in the NAFL group. As shown in Table 1, the NAFLD activity scores (NAS) in the NAFL group for steatosis were 0–3 (n = 0, 23, 22, and 12, respectively); those for lobular inflammation were 0–3 (n = 24, 30, 3, and 0, respectively); and those for hepatocyte ballooning were 0–2 (n = 54, 2, and 1, respectively). The NAS (NAFLD activity score) in NASH group for steatosis were 0–3 (n = 0, 23, 30, and 21, respectively); those for lobular inflammation were 0–3 (n = 0, 38, 31, and 5, respectively); and those for hepatocyte ballooning were 0–2 (n = 1, 46, and 27, respectively). The Brunt classifications of the fibrosis stage in the NAFL group were 0–4 (n = 30, 27, 0, 0, and 0, respectively), while those in the NASH group were 0–4 (n = 2, 37, 10, 23, and 2, respectively).

**Table 1:** Clinical, serological, and pathological characteristics of the NAFLD patients.

	NAFL	NASH	*P* value
N	57	74	
Male (%)	68.4	43.2	<0.01
Age (Yr)	48 ± 15	55 ± 15	<0.01
BMI (kg/m^2^)	28 ± 4	30 ± 6	NS
Plt (10^4^/μL)	22 ± 6	20 ± 7	NS
Albumin (g/dL)	4.4 ± 0.3	4.2 ± 0.4	<0.01
T-bil (mg/dL)	0.8 ± 0.4	0.8 ± 0.3	NS
AST (IU/L)	50 ± 24	67 ± 38	<0.01
ALT (IU/L)	93 ± 67	86 ± 64	NS
ChE (U/L)	379 ± 92	328 ± 121	NS
γ-GTP (IU/L)	97 ± 113	82 ± 59	NS
AFP (ng/mL)	2.4 ± 1.8	3.5 ± 2.7	NS
TG (mg/dL)	174 ± 83	158 ± 79	NS
LDL-C (mg/dL)	131 ± 29	114 ± 39	<0.05
HDL-C (mg/dL)	50 ± 15	45 ± 16	NS
HbA1c (%)	5.9 ± 0.7	6.2 ± 1.8	<0.001
FBS (mg/dL)	106 ± 26	123 ± 45	<0.05
IRI (μIU/L)	20.0 ± 30.5	14.1 ± 11.1	NS
HOMA-IR	5.7 ± 9.3	4.5 ± 4.4	<0.05
cCK18 (U/L)	572 ± 359	927 ± 698	<0.01
M2BPGi (COI)	0.73 ± 0.66	1.24 ± 0.95	<0.001
FIB4 index	1.55 ± 1.4	2.31 ± 1.53	<0.001
APRI	0.60 ± 0.34	0.94 ± 0.69	<0.001
**Pathological findings**			
Steatosis (0/1/2/3)	0/23/22/12	0/23/30/21	
Lobular inflammation (0/1/2/3)	24/30/3/0	0/38/31/5	
Ballooning (0/1/2)	54/2/1	1/46/27	
Fibrosis (0/1/2/3/4)	30/27/0/0/0	2/37/10/23/2	

Data are presented as mean ± SD. P values correspond to the comparison between NAFL and NASH groups. Mann Whitney U tests for continuous factors and Fisher’s exact test for categorical factors were used. Abbreviations: NAFL, non-alcoholic fatty liver disease; NASH, non-alcoholic steatohepatitis.

### AAT and AAT-A3F concentrations in serum from NAFLD patients

**Figure 1:**
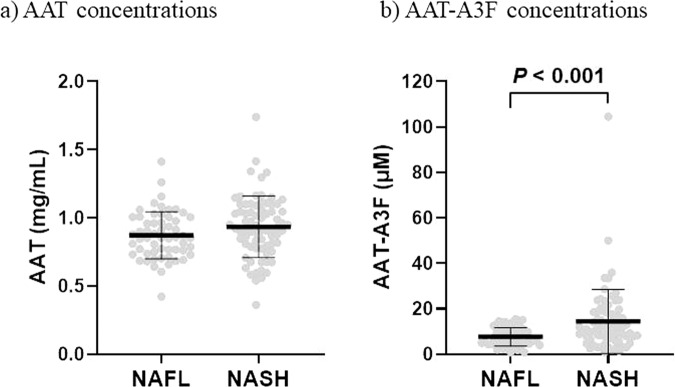
Serum AAT and AAT-A3F concentrations in patients with NAFLD. The vertical axis represents AAT levels in mg/mL and AAT-A3F levels in μM, and the horizontal axis represents the patient groups. Data are shown as mean ± SD. (**A**) The AAT levels were 0.87 ± 0.17 mg/mL for the NAFL group, and 0.93 ± 0.23 mg/mL for the NASH group (n.s.). (**B**) The AAT-A3F levels were 7.63 ± 4.04 μM for the NAFL group and 14.4 ± 14.08 μM for the NASH group (P < 0.001).

Serum AAT concentrations were not significantly different between the NAFL and NASH groups (0.87 ± 0.17 mg/mL in the NAFL group, 0.93 ± 0.23 mg/mL in the NASH group; Fig. 1a). However, serum AAT-A3F concentrations were significantly higher in the NASH group (7.63 ± 4.04 μM) than in the NAFL group (14.40 ± 14.08 μM; P < 0.001; Fig. 1b).

**Figure 2:**
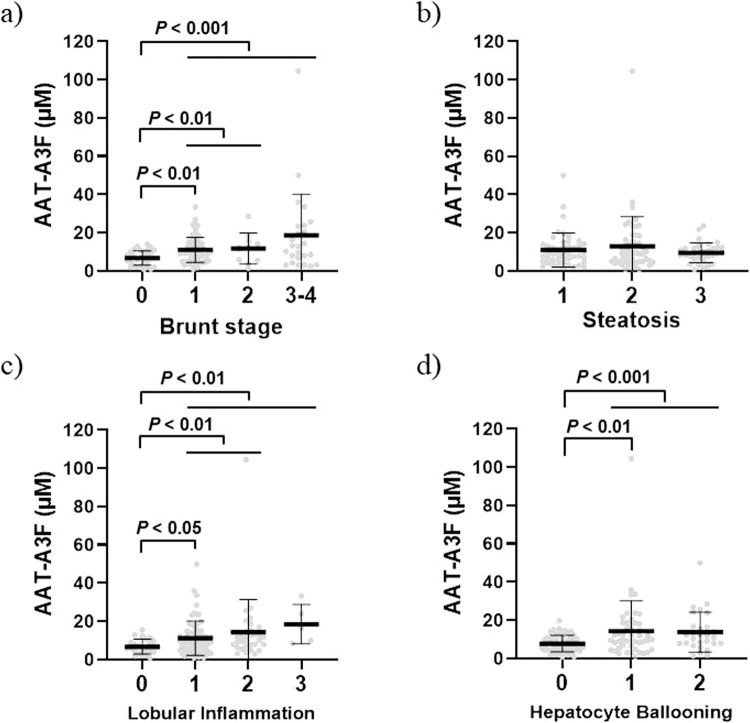
AAT-A3F levels according to the pathological score classification. The vertical axis represents AAT-A3F levels in μM, and the horizontal axis represents the Brunt stage and NAS (based on steatosis, lobular inflammation, and hepatocyte ballooning). Data are shown as mean ± SD. (**a**) The AAT-A3F levels were 6.75 ± 3.73 μM for stage 0, 11.00 ± 6.58 μM for stage 1, 11.66 ± 8.11 μM for stage 2, and 18.55 ± 21.53 μM for stages 3 and 4. (**b**) The AAT-A3F levels were 11.06 ± 8.92 μM for steatosis 1, 12.98 ± 15.46 μM for steatosis 2, and 9.60 ± 5.14 μM for steatosis 3. (**c**) The AAT-A3F levels were 6.74 ± 3.88 μM for lobular inflammation 0, 11.15 ± 9.02 μM for lobular inflammation 1, 14.37 ± 17.03 μM for lobular inflammation 2, and 18.45 ± 10.31 μM for lobular inflammation 3. (**d**) The AAT-A3F levels were 7.80 ± 4.36 μM for hepatocyte ballooning 0, 14.29 ± 15.74 μM for hepatocyte ballooning 1, and 13.77 ± 10.38 μM for hepatocyte ballooning 2.

With respect to the Brunt stage, the serum AAT-A3F levels significantly increased with fibrosis. Serum AAT-A3F levels at stage F0 was 6.75 ± 3.73 μM, that at stage F1 was 11.00 ± 6.58 μM, that at stage F2 was 11.66 ± 8.11 μM, and that at stage F3/4 (with advanced fibrosis) was 18.55 ± 21.53 μM (P < 0.01; Fig. 2a). Furthermore, the relationship between serum AAT-A3F concentration and pathological activity was analysed according to the NAS classification. Serum AAT-A3F levels were not associated with steatosis (S1, 11.06 ± 8.92 μM; S2, 12.98 ± 15.46 μM; S3, 9.60 ± 5.14 μM; Fig. 2b), but significantly increased with lobular inflammation (LI0, 6.74 ± 3.88 μM; LI1, 11.15 ± 9.02 μM; LI2, 14.37 ± 17.03 μM; LI3, 18.45 ± 10.31 μM; Fig. 2c) and hepatocyte ballooning (B0, 7.80 ± 4.36 μM; B1, 14.29 ± 15.74 μM; B2, 13.77 ± 10.38 μM; Fig. 2d).

### Correlation between serum AAT-A3F concentrations and clinicopathological parameters

We analysed correlations between serum AAT-A3F levels and clinicopathological parameters. According to the clinical data, the AAT-A3F levels showed a significant negative correlation with albumin (*r* = −0.252, P < 0.001), ChE (*r* = −0.284, P < 0.001), and TG (*r* = −0.113, P < 0.05), and a significant positive correlation with M2BPGi (*r* = 0.415, P < 0.001), and FIB-4 index (*r* = 0.268, P < 0.01). The pathological data revealed a significant positive correlation between the AAT-A3F levels and lobular inflammation (*r* = 0.314, P < 0.01), hepatocyte ballooning (*r* = 0.285, P < 0.01), and fibrosis (*r* = 0.292, P < 0.001), as shown in Table 2.

**Table 2:** Correlation between AAT-A3F and clinicopathological parameters.

	r	*P* value
Age (Yr)	0.092	NS
BMI (kg/m^2^)	0.066	NS
Plt (10^4^/μL)	−0.172	NS
Albumin (g/dL)	−0.252	<0.001
T-bil (mg/dL)	0.071	NS
AST (IU/L)	0.196	NS
ALT (IU/L)	−0.019	NS
ChE (U/L)	−0.284	<0.001
γ-GTP (IU/L)	0.165	NS
AFP (ng/mL)	0.188	NS
TG (mg/dL)	−0.113	<0.05
LDL-C (mg/dL)	0.087	NS
HDL-C (mg/dL)	−0.136	NS
HbA1c (%)	0.035	NS
FBS (mg/dL)	0.009	NS
IRI (μIU/L)	0.178	NS
HOMA-IR	0.130	NS
cCK18 (U/L)	0.121	NS
M2BPGi (COI)	0.415	<0.001
FIB4 index	0.268	<0.01
APRI	0.236	NS
**Pathological findings**		
Steatosis (0/1/2/3)	−0.012	NS
Lobular inflammation (0/1/2/3)	0.314	<0.01
Ballooning (0/1/2)	0.285	<0.01
Fibrosis (0/1/2/3/4)	0.292	<0.001

The relationship between AAT-A3F and clinicopathological parameters was analysed using Spearman’s R correlations.

### Diagnostic performance of AAT-A3F and other biomarkers for predicting NASH

**Figure 3:**
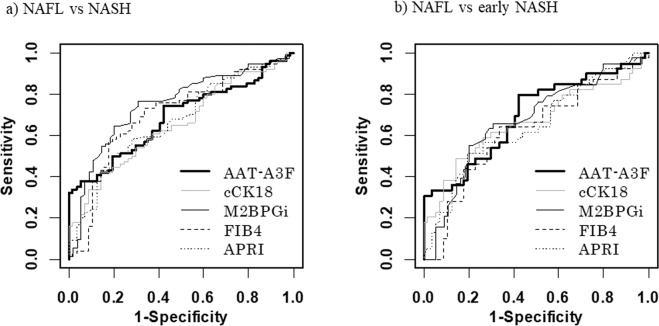
Diagnostic performance of AAT-A3F and other biomarkers for predicting NASH and early NASH. (**a**) ROC curves for diagnosing NASH. (**b**) ROC curves for diagnosing early NASH.

Using ROC analysis, the cut-off value of serum AAT-A3F for NASH diagnosis was set to 14.1 μM. The area under the ROC curve (AUROC) of AAT-A3F was 0.687, and the sensitivity, specificity, PPV, NPV of AAT-A3F were 38%, 95%, 90%, and 54%, respectively. The cut-off value of other markers for NASH diagnosis was set to 977 for cCK18, 0.69 for M2BPGi, 1.44 for FIB4 index, and 0.86 for APRI. The AUROC values of NASH diagnosis were 0.655 for cCK18, 0.749 for M2BPGi, 0.700 for the FIB4 index, and 0.672 for the APRI (Table 3, Fig. 3a).

**Table 3:** Diagnostic performance of AAT-A3F and other biomarkers for predicting NASH.

	AUC	Cut-off	Sensitivity (%)	Specificity (%)	PPV (%)	NPV (%)
AAT-A3F	0.687	14.1	38	95	90	54
cCK18	0.655	977	42	86	79	53
M2BPGi	0.749	0.69	77	69	77	69
FIB4 index	0.700	1.44	73	67	74	66
APRI	0.672	0.86	50	81	77	55

Abbreviations: AUC, area under the curve; NPV, negative predictive value; PPV, positive predictive value; NASH: non-alcoholic steatohepatitis

The cut-off value of serum AAT-A3F for diagnosis of early NASH (Brunt stages 0–1), was set to 7.9 μM in the ROC analysis. The AUROC of AAT-A3F was 0.696, and the sensitivity, specificity, PPV, NPV of AAT-A3F were 79%, 58%, 56%, and 80%, respectively. The cut-off value of other markers for early NASH diagnosis was set to 977 for cCK18, 0.79 for M2BPGi, 1.44 for FIB4 index, and 0.86 for APRI, similar to NASH diagnosis. The AUROC values of early NASH were 0.665 for cCK18, 0.673 for M2BPGi, 0.624 for the FIB4 index, and 0.648 for the APRI (Table 4, Fig. 3b).

**Table 4:** Diagnostic performance of AAT-A3F and other biomarkers for predicting early NASH (Brunt stage S0-1).

	AUC	Cut-off	Sensitivity (%)	Specificity (%)	PPV (%)	NPV (%)
AAT-A3F	0.696	7.9	79	58	56	80
cCK18	0.665	977	49	86	70	71
M2BPGi	0.673	0.79	55	80	66	72
FIB4 index	0.624	1.44	64	67	57	73
APRI	0.648	0.86	51	81	65	71

Abbreviations: AUC, area under the curve; NPV, negative predictive value; PPV, positive predictive value; NASH: non-alcoholic steatohepatitis.

### Gene expression analysis

**Figure 4:**
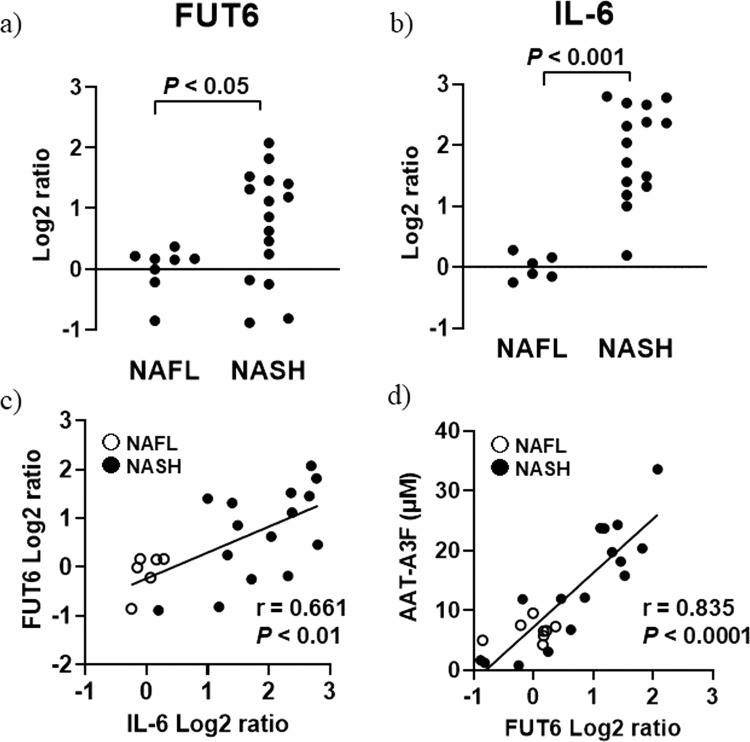
Gene expression analysis. (**a**) The vertical axis represents the FUT6 expression level (log2 scale), and the horizontal axis represents the patient groups. (**b**) The vertical axis represents the IL-6 expression level (log2 scale), and the horizontal axis represents the patient groups. (**c**) The vertical axis represents the FUT6 expression level (log2 scale), and the horizontal axis represents IL-6 expression level (log2 scale). A scatter diagram of FUT6 and IL-6 expression is shown. (**d**) The vertical axis represents the AAT-A3F expression level in μM, and the horizontal axis represents the FUT6 expression level (log2 scale). A scatter diagram of AAT-A3F and FUT6 expression is shown.

Hepatic FUT6 gene expression levels were significantly higher in the NASH group than in the NAFL group (0 ± 0.384 in the NAFL group, 0.747 ± 0.910 in the NASH group (log2 ratio), P < 0.05) (Fig. 4a). IL-6 expression levels were significantly higher in NASH than in NAFL (0 ± 0.202 in the NAFL group, 1.891 ± 0.777 in the NASH group (log2 ratio), P < 0.001) (Fig. 4b). A significant positive correlation was observed between the expression levels of FUT6 and IL-6 (*r* = 0.661, P < 0.01) (Fig. 4c). The serum levels of AAT-A3F strongly correlated with hepatic FUT6 expression levels (*r* = 0.835, P < 0.0001) (Fig. 4d).

## Discussion

In patients with NAFLD, NAFL shows a non-progressive clinical course, whereas NASH is a serious disease with a high risk of both overall and liver-related morbidity and mortality^2–4^. Therefore, differentiating between NAFL and NASH is extremely important in the clinical management of NAFLD patients. Although liver biopsy is still considered a gold standard for differentiating between NASH and NAFL^5^, a non-invasive and reliable diagnostic biomarker is required.

Here, we validated the diagnostic utility of AAT-A3F identified in our previous study^15^. Serum AAT levels were not different between patients with NASH and NAFL, but AAT-A3F was significantly elevated in patients with NASH versus patients with NAFL. In addition, AAT-A3F levels increased significantly not only with hepatic fibrosis, but also with intrahepatic inflammation and hepatocyte ballooning. AAT is a major, liver-derived circulating protein that functions as a natural inhibitor of various serine proteases and is a component of the acute-phase response^16^. During inflammation, the liver synthesises acute phase proteins (APPs), including AAT, which are thought to contribute to the inflammatory response, but also play a central role in limiting local and systemic inflammation^17^. APPs play a dual role in inflammation, and AAT can induce the production of both pro-inflammatory interleukin 1 (IL-1) and anti-inflammatory IL-1 receptor antagonist (IL-1Ra) in peripheral blood mononuclear cells. Other studies have shown that AAT inhibits neutrophil superoxide production^18^, prevents hepatocyte apoptosis^19^, and functions as an endogenous inhibitor of pro-inflammatory cytokine production in whole blood^20^. Thus, AAT is thought to be related to intrahepatic inflammation.

To clarify the mechanism underlying AAT-A3F elevation in NASH, we evaluated gene expression levels in liver biopsy tissues. The expression level of the FUT6 gene, whose protein product is responsible for outer arm fucosylation of glycoproteins secreted from the liver^21^, was significantly higher in NASH than in NAFL. It was previously reported that FUT6 expression was positively regulated by IL-6^22^, and hence, we determined the hepatic expression level of IL-6. IL-6 expression level was significantly higher in NASH than in NAFL, as previously described^23^. A positive and significant correlation between the expression levels of IL-6 and FUT6 was observed, indicating that inflammatory response accompanying IL-6 production in NASH liver can lead to FUT6 upregulation. The serum level of AAT-A3F was strongly correlated with the hepatic FUT6 expression level. Therefore, we conclude that hepatic inflammation caused the elevation in FUT6 expression, resulting in an increase in outer arm fucosylation of AAT produced in the liver.

In this study, the AUROC for NASH diagnosis of AAT-A3F was 0.687, which was higher than cCK18. The apoptosis marker cCK18 was reported to be useful for distinguishing between healthy subjects and those with NAFL, and further between patients with NAFL versus those with NASH^24^. In this study, the diagnostic utility of AAT-A3F was found to be higher than that of cCK18 in our cohort. The AUROC for NASH diagnosis of AAT-A3F was lower than that of M2BPGi and FIB-4 index, however, the AUROC values of AAT-A3F for diagnosing early NASH was higher than that of biomarkers tested in this study. Furthermore, while the cut-off values of cCK18, M2BPGi, FIB-4 index, and APRI for diagnosing NASH and early NASH were very close, the cut-off values in AAT-A3F were nearly doubled. This suggests that AAT-A3F can stratify NASH by the degree of fibrosis. These results may perhaps be because AAT-A3F also reflects liver inflammation and fibrosis. Although liver fibrosis was considered the most important factor for predicting the prognosis of NASH^25^, the mortality rate is reported to increase since mild fibrosis^26,27^. In addition, lobular inflammation has been reported as a risk factor for the progression of liver fibrosis, compared to fatty liver alone^28^. In general, the prognosis of patients with NAFLD is often lower than that of the general population^29^; therefore, it is necessary to intervene at an early stage with heathier diet and exercise. Identifying biomarkers that indicate lobular inflammation and hepatocyte ballooning as well as fibrosis such as AAT-A3F can lead to early diagnosis and treatment of NASH, which helps reduce mortality.

In the previous study, we comprehensively analysed N-glycans of serum glycoproteins in NAFLD patients using the FPG method and found that AAT-A3F could be a useful biomarker for NASH diagnosis. FPG is a useful method for biomarker discovery, but it is not suitable for examinations of many samples, because it requires many steps. Therefore, we have developed a simplified IPG method suitable for the measurement of a large number of samples. Using the IPG method, we verified the usefulness of AAT-A3F in this study. Because the IPG method can be used for any proteins, this method will be applicable to the analysis of glycobiomarkers related to various diseases.

The primary limitation of our study was that the number of patients included was relatively small, and our NAFLD patients were enrolled from a regional liver disease hospital. Therefore, the potential for referral bias cannot be ruled out in our study. Thus, our findings for liver biopsy-proven NAFLD might not represent patients with NAFLD in the general population. Therefore, larger multicentre studies are required to validate our results. The secondary limitation is that we used liver biopsy as the gold standard for assessing the utility of AAT-A3F. This technique has serious limitations including those related to sampling errors and pathological assessment. Therefore, liver biopsy specimens from various facilities were also centrally evaluated by a hepatopathologist in this study. The third limitation is the small sample size that was subjected to gene expression analyses. Although in a multi-centre study, the number of the liver tissue storage are limited, we analysed all possible cases.

**Figure 5:**
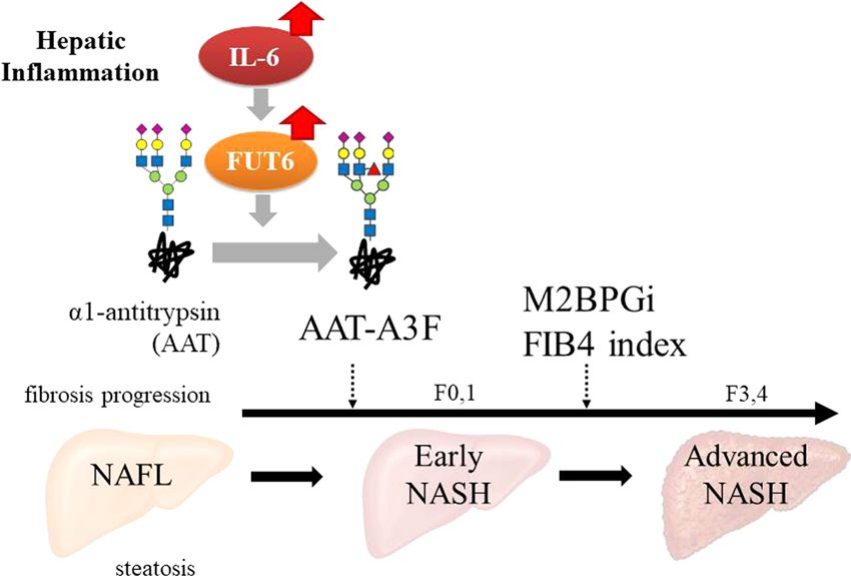
Application of AAT-A3F to clinical diagnosis of NASH. Summarize the findings of this study and show the usefulness of AAT-A3F in NASH diagnosis with illustrations.

In conclusion, this multicentre study revealed that AAT-A3F was especially increased in not only fibrosis, but also pathological inflammation and hepatocyte ballooning in patients with NAFLD, and AAT-A3F was considered useful for non-invasive diagnosis of early NASH. Furthermore, it was beneficial for the diagnosis of NASH by combining AAT-A3F with fibrosis markers such as M2BPGi and FIB4 index depending of progression stage of NASH (Fig. 5).

## Methods

### Study populations

In this study, we included 131 biopsy-proven NAFLD patients from the Hokkaido University Hospital and other affiliated hospitals belonging to NORTE study group. These patients were examined between 2007 and 2017 and underwent a percutaneous liver needle biopsy. Biopsied liver samples were embedded in paraffin blocks following standard procedures and stained with haematoxylin and eosin, Masson’s trichrome stain, and Gitter stain. All biopsy specimens were evaluated by a hepatopathologist blinded to the clinical data. Samples were investigated and quantified based on NAFLD-activity scoring^30^ for steatosis (0–3), lobular inflammation (0–3), and hepatocyte ballooning (0–2). NASH was diagnosed when steatosis, ballooning, lobular inflammation and hepatocyte ballooning of any grade were all present at the same time^31,32^. Individual fibrosis parameters were scored based on the fibrosis stage of the Brunt classification^33^. Early NASH was defined as NASH with no or mild fibrosis (Brunt stages S0–1). Advanced fibrosis was classified as a Brunt stage of 3–4 (S3–4).

The exclusion criteria for this study included daily alcohol consumption (>30 g for men or >20 g for women) and another hepatic disease such as hepatitis B, hepatitis C, hepatocellular carcinoma, autoimmune hepatitis, primary biliary cholangitis, primary sclerosing cholangitis, hemochromatosis, α1-antitrypsin deficiency, Wilson’s disease, or congestive liver disease, according to our previous study^15^. The study protocol complied with the ethical guidelines of the Declaration of Helsinki and was approved by the Institutional Review Board of Hokkaido University Hospital and each participating hospital. Written informed consent to participate in this study was obtained from each patient. This study was registered in the UMIN Clinical Trials Registry as UMIN000025644.

### Collection of clinical data

Clinical data including gender and age were obtained for each patient at the time of liver biopsy. Anthropometric variables (body height and weight) were measured in the standing position, and the body mass index (BMI) was calculated as the weight divided by height in meters squared. Serum biochemical variables (platelet count [Plt], albumin, total bilirubin, aspartate aminotransferase [AST], alanine aminotransferase [ALT], γ‐glutamyltransferase, cholinesterase [ChE], α-fetoprotein [AFP], triglyceride [TG], low-density lipoprotein LDL-cholesterol [LDL‐C], high-density lipoprotein cholesterol, haemoglobin A1c [HbA1c], fasting blood sugar [FBS], and immunoreactive insulin) were measured with a conventional automated analyser. Insulin resistance was evaluated based on the homeostasis model assessment as an index of insulin resistance (HOMA-IR) value using the following equation: HOMA-IR value = fasting insulin (µU/mL) × fasting glucose (mg/dL)/405. Caspase-cleaved cytokeratin 18 (cCK18) and M2BPGi were measured as markers of hepatocellular apoptosis and liver fibrosis, respectively. The formula used for predicting liver fibrosis from non-invasively obtained data was as follows: fibrosis 4 (FIB4) index = age × AST (IU/L) × Plt ( × 10^9^/L)^−1^ × √ALT (IU/L)^−1^, as reported previously^34^. The AST-to-platelet ratio index [APRI] was calculated as AST (IU/L) × AST (upper limit of normal [IU/L])^−1^ × Plt (×10^9^/L)^−1^ ^35^.

### Measurement of tri-sialylated mono-fucosylated tri-antennary glycan (A3F) bound to alpha-1 antitrypsin (AAT)

Serum concentrations of AAT-A3F were determined as previously described^14^. Briefly, AAT was purified from 50 μL of serum using commercially available immune-affinity beads (Alpha-1 Antitrypsin Select, GE Healthcare). The serum AAT concentration was determined by ELISA (Human Serpin A1 DuoSet ELISA, R&D Systems, Minneapolis, MN) with a standard AAT protein (Sigma-Aldrich, A9024). After tryptic digestion of AAT, N-glycans were released with N-glycosidase F. Released glycans were purified and derivatised with *N*^α^-((aminooxy)acetyl) tryptophanylarginine methyl ester using a glycoblotting procedure^36^ and analysed with an Ultraflex II TOF/TOF mass spectrometer. The amount of A3F was quantified by the area ratio using ethyl-esterified A3 (3.8 pmol) as an external glycan. Serum concentrations of AAT-A3F were calculated by dividing the amount of A3F by AAT concentrations of each patient.

### Real-time quantitative PCR analysis

We evaluated gene expression levels in liver tissues from 8 patients with NAFL and 16 patients with NASH. Total RNA was extracted from biopsied liver samples using the AllPrep DNA/RNA/Protein Mini Kit (Qiagen, Venlo, Netherlands). The quantity and quality of the isolated RNA were determined using an Agilent 2100 Bioanalyzer with the RNA 6000 Nano Kit (Agilent Technologies). Total RNA (500 ng) was reverse transcribed using PrimeScript RT Master Mix (Perfect Real Time) (Takara Bio, Shiga, Japan). Real-time quantitative PCR was performed using the Applied Biosystems 7500 Real-Time PCR System. Gene-specific primers were purchased from Takara Bio. Gene expression values were normalised to that of the reference gene, peptidylprolyl isomerase A, and calculated based on the ΔΔCt method^37^.

### Statistical analysis

Statistical analysis was conducted using R software (version 3.5.1). Results are presented as the mean ± standard deviation (SD) for continuous data, and as numbers (percentages) for categorical data. Statistical analysis included descriptive statistics, analysis of variance, Mann–Whitney U test, Fischer’s exact test, and Spearman R correlations. All P values were two-tailed, and the threshold of statistical significance was set at P < 0.05. The diagnostic performance of the markers was assessed by analysing receiver operating characteristic (ROC) curves. The probabilities of true positives (sensitivity), true negatives (specificity), positive-predictive values (PPVs), and negative-predictive values (NPVs) were determined for selected cut-off values, and the AUROC was calculated for each index. Cut-off points were determined based on the optimum sum of the sensitivity and specificity.
